# Representations of personally familiar voices are better resolved in the brain

**DOI:** 10.1016/j.cub.2025.03.081

**Published:** 2025-04-18

**Authors:** Elise Kanber, Clare Lally, Raha Razin, Victor Rosi, Lúcia Garrido, Nadine Lavan, Carolyn McGettigan

**Affiliations:** 1 Department of Speech, Hearing and Phonetic Sciences, UCL, Chandler House, 2 Wakefield Street, London WC1N 1PF, UK; 2 Department Experimental Psychology, UCL, 26 Bedford Way, London WC1H 0AP, UK; 3 Department of Psychology, City St George’s, University of London, Northampton Square, London EC1V 0HB, UK; 4 School of Biological and Behavioural Sciences, Queen Mary University of London, Mile End Road, London E1 4NS, UK

## Abstract

The human voice is highly flexible, allowing for diverse expression during communication,^1^ but presents perceptual challenges through large acoustic variability.^2–11^ The ability to recognize an individual person’s voice depends on the listener’s ability to overcome this within-speaker variability to extract a single identity percept.^2,18^ Previous work has found that this process is greatly assisted by familiarity,^6,9,13^ with evidence suggesting that more extensive and varied exposure to a voice is associated with the formation of a more robust mental representation of it.^4,8^ Here, we used functional magnetic resonance imaging (fMRI) with representational similarity analysis^14^ to characterize how personal familiarity with a voice is reflected in neural representations. We measured and compared brain responses with voices of differing familiarity—a personally familiar voice, a voice familiarized through lab training, and a new (untrained) voice—while listeners identified these voices from naturally varying, spontaneous speech clips. Personally familiar voices elicited brain response patterns in voice-, face-, and person-selective corticesthat showed higher within- and between-speaker dissimilarity, compared with lower-familiarity lab-trained and untrained voices. These findings indicated that representations for the sounds of personally familiar voices are better resolved from each other in the brain, and they align with other research reporting intelligibility advantages for speech produced by familiar talkers.^15–18^ Overall, our findings suggest that extensive and varied exposure to personally familiar voices results in the development of finer-grained representations of those voices, which cannot be achieved via short-term lab training.

## Results

One proposal for how voice identity could be represented in the brain is via stimulus-invariant response patterns that are relatively consistent across different encounters with a given identity (“telling together”) but are distinct from responses to other identities (“telling apart”). Studies of multivariate patterns of brain responses to voices have indeed found evidence that the superior temporal cortex can discriminate between identities, across different vocal stimuli^19,20^ and across modalities (from voices to faces and vice versa^20,21^). These studies focused on how telling different voices apart might be represented in the brain but did not explicitly interrogate the role of within-person variability in shaping representations of voices (or faces). More recently, Lally et al.,^5^ harnessed feature films as sources of naturalistic within- and between-person facial and vocal variability, finding widespread evidence in voice-, face-, and person-selective cortical areas for greater within-person than between-person similarity in brain responses to people (i.e., faces and voices). However, familiarity was not manipulated in that study.

We therefore designed a novel study to address two research questions: (1) do brain representations of voice identities align with a theoretical framework of telling together and telling apart, and (2) how are brain responses to voice identities shaped by speaker familiarity? To do this, we analyzed functional magnetic resonance imaging (fMRI) data from 26 adult participants listening to naturally varying, spontaneous speech recordings from 3 voice identities: a personally familiar voice (Familiar), a voice trained to familiarity via pre-scan training tasks (Lab), and a voice not learned before the scan (New). Using representational similarity analysis (RSA)^14^ of the brain’s response patterns to these voices, we then tested two predictions about the brain’s representation of voice identity. First, we predicted that there would be greater similarity of brain response patterns to the same speaker, compared with response patterns to different speakers (i.e., greater similarity for telling together than telling apart^5,22^). Second, we predicted familiarity-dependent variation in the similarity of within-speaker brain response patterns. Specifically, we expected to see the greatest within-speaker similarity in response to the Familiar voice, in line with behavioral evidence of greater telling together accuracy for familiar voice perception,^6,9,13^ as well as proposed “voice recognition units”^22^ and other models positing abstracted representations of voice identities.^23^

We expected to find representation of voice identities in the temporal lobes, with statistical peaks in the right hemisphere.^19,24–30^ Within this, we expected familiarity effects in the anterior superior temporal sulcus (STS)^31–34^ and person-selective cortical areas that may be implicated in multimodal representations of familiar identities (e.g., precuneus^35,36^). As our hypotheses did not concern higher-order domain-general processes, we restricted our analyses to brain regions defined as voice-, face-, and personselective in previous research on the neural representations of voice, face, and multimodal (voice and face) identities.^5,20^

**Figure 1:**
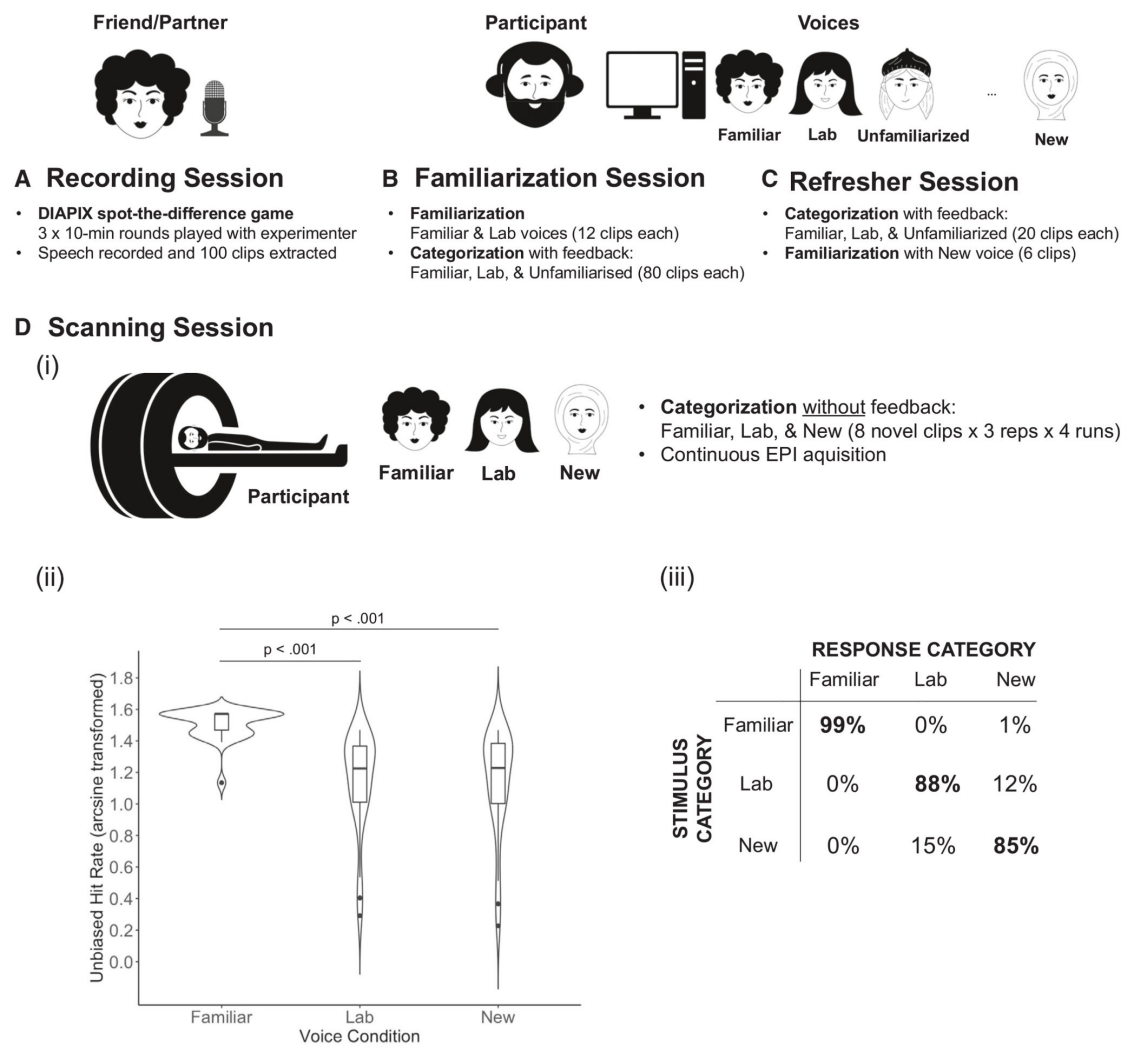
Outline of the study test sessions (A) Recording session: a friend/partner of each participant attended a recording session to generate experimental stimuli. (B) Familiarization session: the participant was familiarized with clips of the Familiar and Lab voice, then completed a voice identity categorization task with feedback. (C) Refresher session: the participant performed one block of the categorization task and was then familiarized with the New voice. (D) Scanner session: (i) the participant underwent four runs of continuous fMRI data acquisition while performing a categorization task without feedback; (ii) in-scanner performance, measured with unbiased hit rates (H_u_) per participant. Violin plots combine a box plot and mirrored density plot. Box plots show the median, 25th, and 75th percentile values. Whiskers extend from the boxplots to the highest and lowest values no more than ± 1.5 times the inter-quartile range; (iii) in-scanner confusions table showing the percentage of trials on which a given presented voice condition (stimulus category) was categorized as the Familiar, Lab, or New voice (response category). Bold indicates the percentage of hits per voice condition. Face drawings: Julia Galuzinskaya/Shutterstock.com.

All participants completed three experimental sessions (Figure 1). In the first familiarization session, participants listened to examples of the Familiar and Lab voices and then completed a three-way forced choice voice identity categorization task with accuracy feedback, including the Familiar voice, the Lab voice, and a third, unfamiliarized voice (mean accuracy [final block]: Familiar 99.6%, Lab 94.7%, unfamiliarized 95.2%). A refresher session, taking place immediately before the scanning session, included shortened training on the identity categorization task with the same three identities (mean accuracy: Familiar 98.4%, Lab 96.0%, unfamiliarized 94.2%). Participants were then briefly familiarized with a novel, previously unheard voice identity (New). In the final fMRI scanning session, we measured neural responses in the context of an explicit voice identity recognition task^37^ to ensure engagement of voice-sensitive processes. Here, behavioral and fMRI data were collected while participants again performed a voice identity categorization task—now without feedback and on *previously unheard* examples of the Familiar voice, the Lab voice, and the New voice (mean accuracy: Familiar 98.9%, Lab 87.6%, New 85.4%). A comparison of unbiased hit rates (H_u_) across the three voice identity conditions showed a significant effect of familiarity (χ^2^_2_ = 40.19, *p* < 0.001), where the Familiar voice was recognized with significantly greater accuracy than both the Lab (estimate = 0.366, t = 6.53, *p* < 0.001) and New (estimate =.376, t = 6.70, *p* < 0.001) voices.

We used RSA^14^ with a searchlight approach^38^ to analyze the brain responses to the three voices within voice-, face-, and person-selective cortical regions of interest.^5,20^ Specifically, for each participant and within each searchlight, we computed brain response patterns to each voice clip, based on the average brain response pattern to that clip across all runs. We then computed the dissimilarities (using 1 − *r* [Pearson correlation]) between the brain response patterns to each pair of clips, resulting in an observed neural dissimilarity matrix (ONDM) for that searchlight. With 8 unique voice clips per 3 voice identities in total, this resulted in a matrix with 276 unique values [i.e., [(24 × 24) − 24]/2]. Per-searchlight ONDMs were entered into partial correlations with hypothetical model representational dissimilarity matrices (RDMs), describing the predicted similarity of neural responses based on our hypotheses. In all cases, the partial correlations quantified the relationship between the brain and the hypothetical model, after controlling for the acoustic dissimilarities between the experimental stimuli (based on their long-term average spectra). In line with convention, below we present and discuss all results in terms of dissimilarity of brain responses, where higher/lower dissimilarity can be interpreted as lower/higher similarity.

### Do brain representations of voice identities align with a theoretical framework of telling together and telling apart?

**Figure 2:**
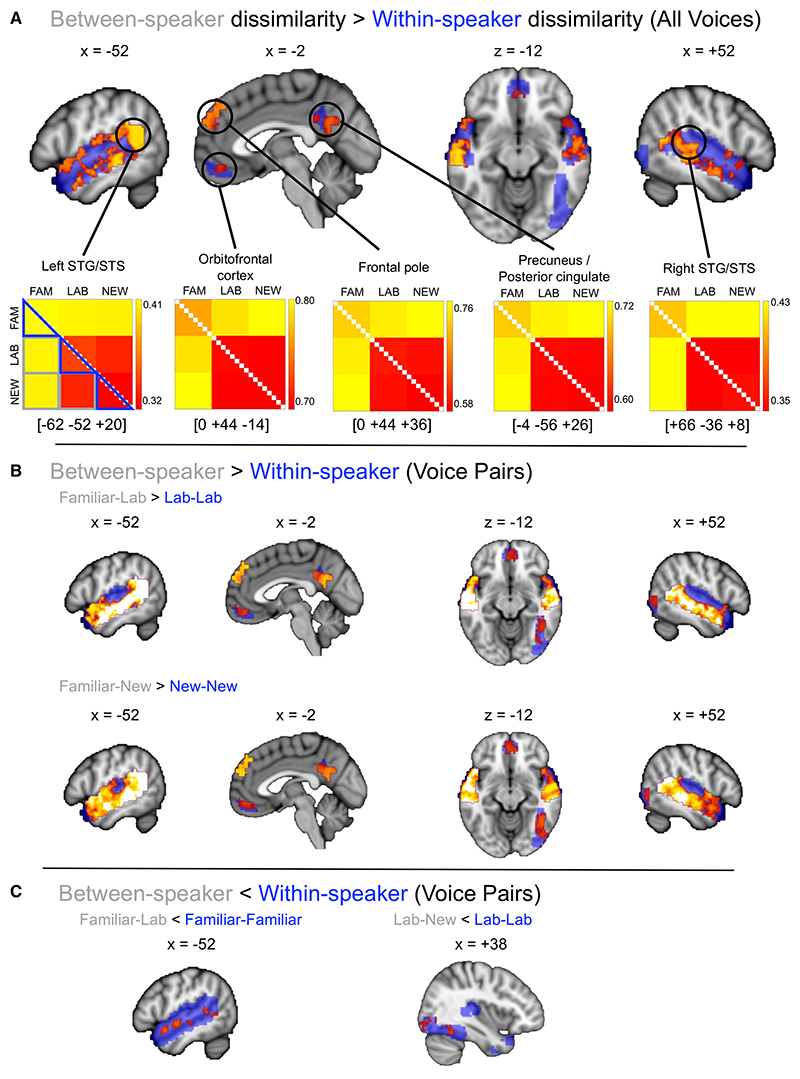
Comparing between-speaker (telling apart) and within-speaker (telling together) dissimilarity in the brain responses to voices (A) Group map of searchlight locations showing significantly greater neural dissimilarity for between- than within-speaker comparisons, across all voice conditions (z > 1.96, with threshold-free cluster enhancement (TFCE) correction). (B) Group maps of searchlight locations showing significantly greater neural dissimilarity for between- than within-speaker comparisons, for selected pairwise voice comparisons (z > 1.96; with TFCE correction; see also Table S1). (C) Group maps of searchlight locations showing significantly greater neural dissimilarity for within- than between-speaker comparisons, for selected pairwise voice comparisons (z > 1.96; with TFCE correction; see also Table S1). Blue shading indicates the searchlight mask of face-, voice-, and person-selective brain regions of interest. Observed neural dissimilarity matrices (ONDMs) illustrate group mean dissimilarity values (1 − Pearson’s correlation) per voice comparison (e.g., Familiar-Lab, New-New), where red indicates the minimum dissimilarity and yellow the greatest dissimilarity within a 100-voxel searchlight volume centered on the location indicated by the coordinates. Coordinates are shown in Montreal Neurological Institute (MNI) stereotactic space; gray squares and blue triangles on the matrices outline the comparisons included in each analysis. Peak center voxels were identified from uncorrected group searchlight maps. STG, superior temporal gyrus; STS, superior temporal sulcus. See also Table S1.

Our first analysis tested a telling together and telling apart framework for representing voice identity, using a model predicting greater dissimilarity of brain responses for between-speaker than within-speaker comparisons. We found evidence for significantly greater between-speaker than within-speaker dissimilarity across our searchlight mask, excluding most of the primary auditory cortex and adjacent portions of the superior temporal gyrus (STG) bilaterally, as well as face-selective right fusiform and occipital gyri (Figure 2A; Table S1). Pairwise follow-up analyses revealed familiarity-dependent profiles, now also implicating the face-selective occipitotemporal cortex: specifically, greater between- versus within-speaker dissimilarity was observed only for between-speaker comparisons that implicated the Familiar voice (Familiar-Lab and Familiar-New), when contrasted with within-speaker comparisons that implicated the less familiar Lab and New voices (i.e., Lab-Lab or New-New; see Figure 2B and Table S1). However, there was no evidence for greater between- than within-speaker dissimilarity when analyzing responses to the Lab and New voices only. These results suggest a more complex picture of telling apart and telling together than hypothesized: as the representative matrices in Figure 2 show, neural responses to the Familiar voice tended to generate the highest dissimilarities within our regions of interest, for both within- and between-speaker comparisons.

There was some evidence for significant *negative* correlations with some hypothetical models, where within-speaker comparisons generated greater dissimilarity in brain responses than between-speaker comparisons. Again, these effects trended with familiarity: multiple clusters in left and right superior temporal cortices showed significantly greater dissimilarity of within-speaker brain responses across familiar voice stimuli than dissimilarity of between-speaker comparisons with the less familiar lab-trained voice, while clusters in right fusiform and inferior occipital gyrus similarly showed greater within-speaker dissimilarity for the lab-trained voice than dissimilarity ofbetween-speaker comparisons with the least familiar, new voice (Figure 2C; Table S1). Taken together, these results suggest that while telling together and telling apart offer a useful framework for describing the behavioral correlates of naturalistic voice identity perception, where telling together selectively benefits from familiarity, this is not directly reflected in the responses of voice-, face-, and person-selective brain regions to voices of varying familiarity.

### Are brain response patterns to individual voice identities shaped by speaker familiarity?

**Figure 3:**
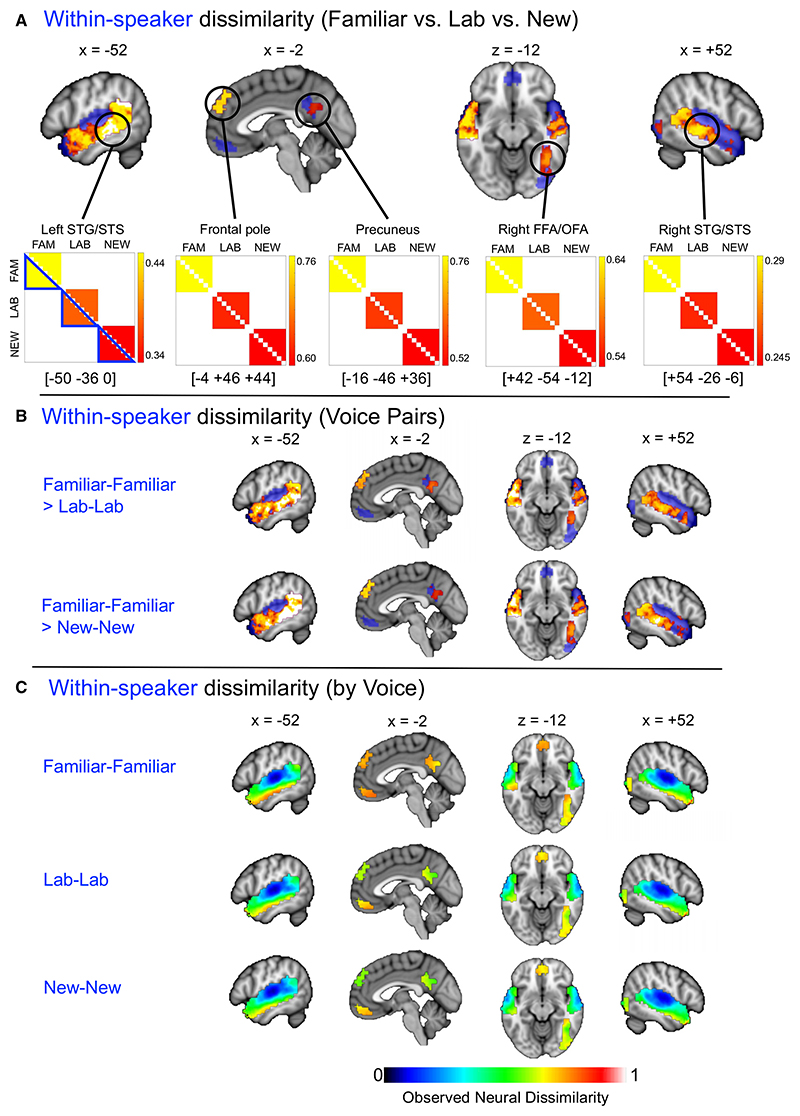
Comparing within-speaker (telling together) dissimilarity in the brain responses to voices of differing familiarity (A) Group map of searchlight locations showing a significant effect of voice condition (Familiar, Lab, New) (z > 1.96, TFCE correction; see also Table S2) on neural dissimilarity. (B) Group maps of searchlight locations showing significantly greater neural within-speaker dissimilarity for the Familiar voice compared with the Lab and New voices, respectively (z > 1.96, TFCE correction). Blue shading indicates the searchlight mask of face-, voice-, and person-selective brain regions of interest. The comparison of the Lab voice with the New voice is not shown as there were no suprathreshold clusters. (C) Group searchlight maps of mean neural dissimilarity for within-speaker comparisons, for each voice condition. Representational dissimilarity matrices (RDMs) illustrate group mean dissimilarity values (1 − Pearson’s correlation) per voice comparison, including only within-speaker comparisons, where red indicates the minimum dissimilarity and yellow the greatest dissimilarity within a 100-voxel searchlight volume centered on the location indicated by the coordinates. Coordinates are shown in Montreal Neurological Institute (MNI) stereotactic space. Peak center voxels were identified from uncorrected group searchlight maps. STG, superior temporal gyrus; STS, superior temporal sulcus; FFA, fusiform face area; OFA, occipital face area. See also Table S2.

To test for effects of familiarity on telling together, we compared within-speaker dissimilarities in brain response patterns with a model predicting increasing within-speaker dissimilarity with decreasing voice familiarity (i.e., Familiar < Lab < New). This revealed significant effects in all voice-, face-, and person-selective regions of interest, except for the primary auditory cortex and adjacent portions of STG, orbitofrontal cortex, and right inferior occipital cortex (Figure 3A; Table S2). Here, against our predictions, we found a negative correlation between brain responses and the model: the Familiar voice generated the greatest within-speaker dissimilarity on average, with responses to the Lab and New voices showing significantly lower and statistically equivalent within-speaker dissimilarity (Figure 3B; Table S2). That is, participants recognized the Familiar voice with greater accuracy while showing *better-resolved* brain responses to examples of this voice, compared with lower recognition accuracy and within-speaker resolvability for the Lab and New voices. Interestingly, while we observe this effect of familiarity along the length of STS in both hemispheres, the raw dissimilarity values for within-speaker comparisons (which underlie the results in Figures 3A and 3B) show an anterior-going hierarchy of processing: within-speaker brain response patterns to individual stimuli increased in their mutual dissimilarity with increasing distance from the primary auditory cortex, and this profile was most pronounced for the familiar voice (Figure 3C).

## Discussion

The current study offers important theoretical insights on how the voices of other people are represented in the human brain. Across all analyses, responses to the personally familiar voice showed higher within- and between-speaker dissimilarity, suggesting that greater familiarity is reflected in better-resolved representations of a voice across different utterances. Brain responses became increasingly resolved from each other along the auditory processing hierarchy^39–43^ and to a greater extent for the personally familiar identity than for less familiar speakers, complementing reports of striking familiarity advantages for speech intelligibility,^15–18^ which are underpinned by more robust speech representations in the superior temporal cortex.^44^ Familiarity effects in person- and face-selective regions within our study potentially reflect engagement of extended knowledge when listening to a familiar voice—beyond speech comprehension, listeners may make finer-grained inferences about the speaker’s appearance, mood, and intentions for each specific utterance.^45–47^

Some regions of interest showed no statistically significant effects: notably, bilateral primary auditory cortices and their immediate surrounds, which in general exhibited very low dissimilarity of brain response patterns across voice stimuli (Figure 3C). This aligns with existing models of voice processing^22,35^ proposing that voice structural and identity-related cues are extracted at later stages of the auditory processing hierarchy. Similarly, findings in face-selective regions of the inferior occipito-temporal cortex implicated the anterior portions of the fusiform gyrus more than the posterior occipital cortex, reflecting the recent finding that the fusiform face area (FFA) represents higher-order information about faces (e.g., gender, traits) while the occipital face area (OFA) represents image-based information.^48^ We note, however, that while we assume that our results reflect higher-order aspects of voice perception (having partialled out basic stimulus acoustics), our study was not designed to establish the informational content of voice representations.

Other areas within our searchlight regions were somewhat inconsistently implicated in the results. Against the overall trends, scattered clusters in the OFA/FFA and (mainly left) superior temporal cortex exhibited effects of *greater* within-speaker than between-speaker dissimilarity for some voice pairs (Figure 2C). These effects should be interpreted in the context of our overall finding that a telling together and telling apart account is likely an insufficient framework for capturing how distinct voice identities are represented in the brain. More consistent was the overall greater resolution of brain responses to the familiar voice, where we found that the person-selective orbitofrontal cortex may be relatively less sensitive to voice familiarity than the other person-selective regions (Figure 3).

Although participants spent three experimental sessions listening to the lab-trained voice and recognized it with high accuracy, the lab-trained voices showed lower within-speaker and between-speaker dissimilarity in brain response patterns, compared with personally familiar voices, and were recognized with significantly lower accuracy during the in-scanner behavioral task. Indeed, the in-scanner drop in recognition accuracy for the lab-trained voice reflects the vulnerability of recognizing a weakly familiar voice^4^ when presented with previously unheard examples, in the context of a new competitor voice and in a noisy scanner environment. The profiles of brain response pattern dissimilarities for the lab-trained voice were also minimally distinct from the untrained new voice, and these two voice identities were categorized with equivalent accuracy in the scanner—although accurate categorization of the new voice can reflect both emergent familiarity and correct rejection of that voice from the other categories (i.e., “It’s not my partner/friend or the voice I’ve learned, so it must be someone else”), limiting our interpretation. Nonetheless, these observations further suggest fragility in the representation of the lab-trained voice in relation to the personally familiar identity. This is important when considering how “familiarity” is operationalized for use in voice identity research: near-ceiling accuracy of voice recognition, following brief training, may be built upon ill-formed representations that generalize poorly to new listening situations. Indeed, voices learned from different amounts of exposure that can be identified with equivalent accuracy still show exposure-dependent levels of familiar-speaker intelligibility benefits.^49^ Modifications of the current study design could include two or more voices per familiarity level, thus allowing for telling apart versus telling together models to be tested at a matched level of familiarity. Similarly, including multiple lab-trained voices learned from varying amounts and types of exposure would allow us to more clearly establish how the resolution of responses to within-speaker variability emerges with increasing familiarity.

Overall, this study challenges the notion that familiar person recognition is underpinned by a neural framework in which between-person variability must exceed within-person variability,^50^ while showing that being more familiar with a person’s voice allows the listener to encode and represent that speaker’s utterances with greater distinction. The latter finding speaks against a mechanism for voice identity recognition purely based on stimulus-invariant voice recognition units^22^ or reference patterns,^23^ instead suggesting that familiarity may be underpinned by neural representations incorporating more, rather than less, detail about how a voice sounds across variable stimuli. Indeed, previous behavioral research has shown that under certain circumstances, greater variability of exposure benefits voice identity learning.^8^ Further, while there is evidence that listeners may learn voice identities by extracting summary statistics of voice patterns (e.g., acoustic averages), memory for variable exemplars is not discarded in the learning process.^51^

It is possible that the brain’s mechanism for recognizing voice identities combines familiar voice pattern matching with representations of learned within-speaker variability. Previous neuroimaging studies reporting sensitivity to voice identity and familiarity in the univariate magnitude of responses in the (right) anterior temporal cortex^28,31–33^ analyzed the brain’s averaged response to voice identities, thus potentially capturing the activation of more robust voice averages or reference patterns for more familiar voices^45^—an exploratory univariate analysis of our data also shows greater responses to the familiar voice, compared with the other two identities, in bilateral anterior temporal lobes (Figure S1; Table S3). However, if representations incorporate the learned variability of a voice, it follows that more detailed representations of personally familiar voices (with which listeners typically have more varied experience) may simultaneously generate greater variability of brain response patterns, as different exemplars of a voice will match with different aspects of its stored variability. Our current RSA approach emphasized the (dis)similarity of responses to individual voice stimuli and thus more likely captured this aspect of voice identity representations. Taking these findings together, we suggest that familiar voice representations may encode within-speaker variability *in addition* to any abstracted familiar voice pattern, rather than instead of it.

## Resource Availability

### Lead contact

Further information and requests for resources and reagents should be directed to and will be fulfilled by the lead contact, Carolyn McGettigan (c.mcgettigan@ ucl.ac.uk).

### Materials availability

This study did not generate new unique reagents.

## Star⋆Methods

### Key Resources Table

**Table:** 

REAGENT or RESOURCE	SOURCE	IDENTIFIER
Deposited Data		
OSF Project: Representations of personally-familiar voices are better resolved in the brain	Center for Open Science	http://doi.org/10.17605/OSF.IO/QRZWG
Software and algorithms		
Gorilla Experiment Builder	Cauldron, UK	https://www.gorilla.sc
Matlab	The Mathworks, US	https://www.mathworks.com/products/matlab.html
Psychtoolbox3	Psychtoolbox	https://psychtoolbox.org/
R	The R Project for Statistical Computing	https://www.r-project.org/
Python	Python Software Foundation	https://www.python.org/
*lme4 emmeans* SPM12	Douglas Bates et al. Russell V. Lenth UCL, UK	https://github.com/lme4/lme4/ https://CRAN.R-project.org/package=emmeans https://www.fil.ion.ucl.ac.uk/spm/software
AFNI CoSMoMVPA Mango	Robert W. Cox and others Nikolaas N. Oosterhof, Andrew C. Connolly, CoSMoMVPA contributors Research Imaging Institute, UTHSCSA	https://afni.nimh.nih.gov/ https://www.cosmomvpa.org/ https://mangoviewer.com/
Other		
DIAPIX headphone & microphone Scanner headphones Scanner projector Scanner Button Box	Beyerdynamic DT297PV headsets Sensimetrics S14 EPSON Nata Technologies	
librosa	McFee et al. ^52^	https://librosa.org/

## Experimental Model And Study Participant Details

### Participants

Twenty-seven adult participants completed all the behavioural and fMRI sessions. All were native speakers of British English and aged between 18 and 50 years old at the time of the scan. Data from 1 participant were excluded following the scan due to issues with fMRI acquisition (inadequate slice positioning) and with in-scanner task performance (9% timeouts on response trials). The final sample for data analysis therefore included 26 participants (19 female, 5 male, 1 non-binary, 1 agender; mean age 26.1 years; 4 left-handed). Ethical approval was obtained from the ethics chair of the Birkbeck-UCL Centre for Neuroimaging (BUCNI) within the UCL Division of Psychology and Language Sciences (Project ID: fMRI/2019/005). All participants provided informed consent before completing any of the recordings or tasks. Participants were paid £6 for the voice recording session and an additional £19 for taking part in the additional behavioural and scanning sessions.

## Method Details

### Stimuli

#### Familiar Voices

In the behavioural and scanning sessions, all 26 participants listened to a familiar speaker who was personally known to them. The initial recruitment strategy was to recruit participants in familiar pairs, where each member of the pair would provide the personally-familiar voice recordings for the other. However, in order to match the personally-familiar voices with the lab-trained and unfamiliarized/new identities used in the experimental tasks, all personally-familiar voices were required to speak with a Southern British English accent, and it was not possible to find enough pairs where both members met this requirement; some recruited participants were also found to be ineligible for the scanning experiment due to MR contraindications.

The final dataset included 8 mutual pairs (i.e. where each participant provided personally-familiar voice recordings for the other, and both completed all experimental sessions). Three of these 16 participants provided personally-familiar voice recordings for a further 5 participants (2 females each heard by 2 additional participants; 1 male heard by 1 additional participant). Finally, 4 participants provided voice recordings only, for a further 5 participants (1 female heard by 3 participants, 3 females each heard by 1 participant) and were not included in the experimental data. In total, there were 21 unique personally-familiar voices in the study (16 female-sounding, 5 male-sounding).

Eight personally-familiar voices were the romantic partner of participant, and the remainder were friends and colleagues. Participants on average reported knowing their familiar partner for 6.9 years (range 3 months − 23 years; median 5.5 years), and speaking with them for 8.6 hours per week (range 30 mins − 30 hours; median 4.5 hours).

#### Lab and New Voices

All voices encountered by each participant in the behavioural and in-scanner tasks were matched in regional accent (Southern British English) and apparent sex (i.e. female-sounding or male-sounding). For each participant, 4 voice identities were needed: 1 personally-familiar voice (labelled with the speaker’s proper forename), 1 lab-trained voice (labelled “Alex”), and 2 unfamiliar voices (1 labelled as “someone else” for the training and refresher sessions; 1 labelled as “Charlie” for the scanning session). Three female-sounding and 3 male-sounding voices, selected from the LUCID corpus,^53^ were assigned to the lab-trained and unfamiliar-speaker roles, with counter-balancing across participants. In total, 27 unique voice identities were heard in the study (19 female-sounding, 8 male-sounding); there were 23 completely unique combinations of the 4 voice identity conditions (personally-familiar, lab-trained, unfamiliar, new), including 10 different combinations of the lab-trained and new voices.

#### Stimulus Recording and Preprocessing

Voice recordings of participants and their personally-familiar partners producing naturally-varying, spontaneous speech were obtained via the DIAPIX task.^53^ To minimise familiarity with the speech content the participants would hear in the behavioural and fMRI sessions of our study, we paired all participants and their partners with an experimenter for the recording session, instructed them not to discuss the DIAPIX task with their partner after the recording session, and where possible assigned different picture sets to each participant within a pair.

The task required a pair of players, who sat in separate sound-attenuated chambers, to engage in an interactive “spot the difference” game. Within each of 3 rounds, each player saw one of the images from a pair of pictures, and the players’ joint goal was to locate all 12 differences between the pictures via spoken discussion. Each participant wore Beyerdynamic DT297PV headsets fitted with cardioid microphones to enable discussion and enabling each voice to be recorded into a single channel without interference from the other player. Speech was recorded and digitised at a sampling rate of 44100Hz. Both participants were required to click with their mouse at the location of each difference; these data were not analysed. Each round lasted as long as it took to find all 12 differences, or until a 10-minute timer ended.

A semi-automated pipeline was then used to identify and preprocess 100 audio stimuli per voice (i.e. all the required personally-familiar, lab, unfamiliar, and new voice identities) for use in the behavioural training and fMRI sessions. First, within each individual voice recording (i.e. one speaker), a script written in R^54^ identified and extracted periods of non-silence lasting 2-3 seconds and containing a maximum pause of 0.5 seconds. These were manually inspected to retain clips containing coherent spoken phrases and exclude unsuitable tokens. Clips of >3 seconds were also retained and manually trimmed to under 3 seconds where needed to complete the target number of 100 stimuli. The 100 clips were then amplitude normalised (root-mean-square) for inclusion in the experimental tasks. For the Lab and New voices, as well as the other unfamiliarized voices used in training, DIAPIX recordings were obtained from the LUCID corpus^53^ and preprocessed following the same pipeline.

A second R script was used to select the 9 longest clips from each set of 100, from which 8 were chosen for inclusion in the fMRI session and the 9^th^ was returned to the set. This was done to ensure that the in-scanner clips would provide robust neural responses. A third R script selected 4 clips per DIAPIX round for each voice to make a total of 12, which were combined into 2 sequences of 6 clips each (labelled A and B) for use as familiarisation stimuli. Finally, an R script converted all experimental stimuli − 2 familiarisation sequences (12 clips total) plus 88 individual clips − to MP3 format for inclusion in the experimental tasks.

The fMRI session voice clips from one of the personally familiar voice identities are available as open data (Open Science Framework: http://doi.org/10.17605/OSF.IO/QRZWG).

### Procedure

#### Behavioural training and refresher sessions

Before the scanning session, participants were invited to complete an online familiarisation training experiment on the Gorilla Experiment Builder platform.^55^ They were encouraged to complete the study using their own computer and headphones, in a quiet environment with no distractions. Participants were introduced to the Familiar and Lab voices by listening to the 6-clip familiarisation sequences per voice, in the order A-B-A (i.e. 18 clips, with 6 clips each repeated once). The Familiar voice was introduced first, with the forename of the participant’s personally-familiar partner, followed by the Lab voice, which was introduced as “Alex”. Participants were encouraged to listen carefully to each voice and try to memorise how it sounded. The participant then performed a voice identity training task including the Familiar voice, the familiarised Lab voice, and one of the two remaining sex-matched unfamiliar voice identities. The training took the form of a 3-alternative forced-choice task, where on each trial the participant was presented with one voice clip and had to choose the identity of the speaker from the three onscreen options “[Familiar partner’s name]”, “Alex”, and “Someone else”. There was no time limit on responses. Feedback was provided on each trial: a correct response was followed with an onscreen green tick, while an incorrect response was first indicated by an onscreen red X and a new screen providing the correct answer (e.g. “Not quite! The **correct** answer was: Alex”). The task was divided into 4 blocks, each containing 20 unique trials per voice in a random order, for a total of 80 trials per voice. To prepare participants for the MRI experiment, the left-to-right assignment of the onscreen response options was different in each block. At the end of each block participants were shown their accuracy during that block, as a percentage. They had the opportunity to have a small break before manually proceeding with the next block.

On the day of the scan, participants completed a refresher training experiment in a quiet room at the Birkbeck-UCL Neuroimaging Centre. The experiment was run on Gorilla, using a MacBook Air laptop and headphones. The procedure was similar to the previous training session, beginning with the same familiarisation of the Familiar and Lab voices and continuing with a forced-choice categorisation of the 3 voice identities with feedback, but this time using only one block of the familiarisation training task (i.e. 20 clips per voice). Following this refresher task, the participants completed a short task in which they received the instructions for the MRI experiment, including a description of the in-scanner response button box and the assignment of buttons to responses, plus a warning that they would not receive task feedback in the scanner. Finally, the participant was informed that in the scanner they would hear their familiar partner’s voice, “Alex”, and a new voice labelled “Charlie”. The task ended with one familiarisation sequence (6 clips) in the voice of “Charlie”. Importantly, participants received no training to recognise the voice of “Charlie” before they entered the scanner, with listening-only familiarization being provided to simply ensure that the participants would not be confused by the presence of this new voice in the scanner task.

The median gap between the familiarisation training and the refresher training / MRI session was 1 day (range: 0 - 31 days).

#### MRI session

The MRI session task comprised 4 functional runs of continuous data acquisition. Within each run, participants performed a voice identity categorisation task programmed in MATLAB (The Mathworks, Natick, MA) using the Psychtoolbox extension.^56^ Each experimental trial began with audio presentation of a voice stimulus (lasting 2000-3000ms; jittered onset with mean = 375ms and std = 125ms), followed by a brief visual fixation cross (500ms) and a visual response prompt (“Whose voice did you hear?” with 3 options displayed left to right onscreen). Participants had 2000ms to provide a response via a button box (Nata Technologies, Coquitlam, Canada), where the buttons beneath their index, middle, and ring fingers corresponded to the left-to-right onscreen arrangement of the response labels. No feedback was given. After the response window, the participant saw a fixation cross for a jittered interval of 250-1000ms. Each run included 96 trials (maximum duration 6000ms), comprising 72 experimental trials (3 voices x 8 stimuli x 3 repetitions each) and 24 null trials (fixation only; mean duration = 5000ms). Stimulus order was pseudorandomised within each run, and the left-to-right assignment of response options was randomised between runs.

Audio stimuli were delivered at a comfortable volume using MR-compatible earbuds (S14; Sensimetrics, Malden, MA). Visual displays were projected (Seiko Epson Corporation, Shinjuku, Japan) to a screen in the scanner bore. The total duration of scanning session was approximately 1 hour per participant.

## Quantification And Statistical Analysis

### Statistical analysis of behavioral data

All of the behavioural data from the familiarisation training, refresher training, and in-scanner tasks was analysed in RStudio (Version 2023.12.1.402).

For the familiarisation and refresher training, responses were coded as correct or incorrect per trial. Mean accuracy was then calculated as a percentage per voice condition (Familiar, Lab, unfamiliar) per participant. Group performance was summarised using means and standard deviations per voice and task. Refresher session data were not recorded for 1 participant due to them completing the task in preview mode on Gorilla.

For the in-scanner task, trial-wise data were coded in terms of accuracy (1 = correct, 0 = incorrect), whether a timeout occurred, and the selected response category (Familiar, Lab, or New). Overall accuracy per voice condition (Familiar, Lab, New) and per participant was calculated as a percentage, and group performance was summarised with means. To statistically compare performance across the three conditions, data per voice condition were summarised as unbiased hit rates (H_u_ scores) using the following formula (using the “Alex” voice as the example):

(Hits("Alex")/Total"Alex"trials)x(Hits("Alex")/Total"Alex"responses)

H_u_ scores were arcsine transformed and entered into a linear mixed model, which was estimated using the *lme4* package^57^ in R with voice condition as a fixed effect and participant as a random intercept:

$$H_{u}\sim Condition+(1|ppid)$$

Significance of the fixed effect of Condition was estimated using ANOVA to compare the full model with a model lacking the fixed effect. Post-hoc pairwise comparisons with Bonferroni correction were performed using the *emmeans* package.^58^

### Imaging Methods

MRI data were acquired on a 3T Siemens MAGNETOM Prisma scanner with a 32-channel head coil (Siemens Healthcare, Erlangen, Germany). EPI data were collected using x4 multiband acceleration^59,60^ with no in-plane acceleration (TR = 1000 ms, TE = 35.2 ms, flip angle = 60 deg., slice tilt = 35 deg., phase-encoding direction = A-P, bandwidth = 2620 Hz/Px, echo spacing = 0.56 ms, excite pulse duration = 4060 us; 48 interleaved slices, slice thickness = 2.0mm, in-plane resolution = 2.0 mm). Two phase-encode reversed volumes were acquired per run for unwarping. Each participant also underwent a GRAPPA-accelerated, T1-weighted MPRAGE anatomical MRI scan (TR = 2.3 seconds, TE = 2.98 ms, 208 sagittal slices, slice thickness = 1.0mm, resolution = 1.0 mm).

### Imaging pre-processing

The fMRI data were preprocessed using SPM12 (Version 7771) and AFNI (Version AFNI_23.3.07). After the first 5 functional volumes were discarded to account magnetic saturation effects, the functional and anatomical images for each participant were manually aligned with the origin. The EPI data were then unwarped in AFNI using the phase-encode reversed images. In SPM12, the images were then realigned, co-registered with the anatomical image, and normalised to the MNI template using parameters generated via the segmentation of the anatomical image.

For each participant, a univariate general linear model was constructed and estimated in SPM12 for use in Representational Similarity Analysis. All experimental and null trial onsets, as well as trial responses, were modelled as instantaneous events and convolved with the canonical haemodynamic response function. The 24 unique experimental items were modelled within individual regressors per run (i.e. 24 voice stimuli containing 3 events each). All 24 null events were modelled within a single regressor per run. Two further regressors modelled the timepoint of correct and incorrect task responses, respectively. For each timeout trial where no response was given, the onset was defined by adding the mean response time across all experimental trials to the timepoint at which the response screen appeared. Movement parameters calculated during realignment were also modelled per run, in six regressors of no interest. Contrast estimate maps were then calculated for each experimental item versus the null baseline (averaged across all runs). Finally, a T map containing the 24 contrast volumes (24 x item > baseline) was saved per participant for use in the multivariate analyses.

### Imaging design

#### Representational Similarity Analysis

Representational Similarity Analysis (RSA) was performed using a searchlight approach within the CoSMoMVPA toolbox^61^ in MATLAB. Specifically, we extracted neural response patterns to between- and within-voice identity comparisons, and compared these with hypothetical models of “telling together and “telling apart” while accounting for acoustic properties of the stimuli.

Searchlight volumes comprised 100 voxels were constructed around each voxel in a pre-defined mask that comprised functional regions of interest previously identified as voice-, face-, and person-selective in a separate study^20^ (see also RSA searchlight mask below). For each searchlight volume, an observed neural dissimilarity matrix (ONDM) was then generated for each participant: Taking the participant’s item-wise T contrast maps (24 x Item > Baseline, averaged across runs) as input, neural response patterns for each item were defined as the T values across all 100 voxels within the searchlight. Neural dissimilarity values for all possible pairwise comparisons of the 24 items (i.e. ((24 x 24) - 24) / 2 = 276 unique comparisons) were then calculated as 1 minus the Pearson’s correlation (1 − *r*) between the response patterns for each pair. Due to concerns about the effects of mean-centering on the relationships between conditions for multivariate correlation analyses,^62,63^ we did not employ mean-centering before calculating dissimilarity values.

RSA proceeded as follows: For each participant and each tested hypothetical model, a partial Spearman correlation was performed per searchlight volume between the ONDM and the hypothetical model representational dissimilarity matrix (RDM; see hypothetical model representational dissimilarity matrices (RDMs)), while controlling for an acoustic model RDM (see acoustic model representational dissimilarity matrices (RDMs)). The output correlation coefficients were Fisher-to-z transformed to enable later comparisons across participants. The partial correlation procedure thus resulted in a brain map of Fisher-transformed correlation values at each voxel in the searchlight mask, where the magnitude of correlation values expressed how well the hypothetical model RDM characterised the observed neural dissimilarity in response to the different stimuli and voice identities.

To test the statistical significance of neural representations, all participant correlation maps per hypothetical model were analysed at group level via voxel-wise one-sample t-tests to compare correlation values with 0. This produced a group-level brain activation map of corresponding z-scores per tested model. Statistics were adjusted for multiple comparisons using threshold-free cluster enhancement (TFCE^64^) with 10,000 iterations. To test for both positive and negative brain-model relationships, the TFCE-corrected maps were finally voxel-wise thresholded at both z = +1.96 and z = -1.96, respectively. This threshold corresponds to p <.05 after correction for multiple comparisons. Due to the possibility of multiple maximum values within TFCE-corrected maps, the uncorrected group maps were used to identify peak voxels for visualisation of ONDMs at those locations. Brain data were visualised and anatomically labelled using Mango (Research Imaging Institute, UTHSCSA). All group searchlight maps are available as open data (Open Science Framework: http://doi.org/10.17605/OSF.IO/QRZWG).

#### Hypothetical model Representational Dissimilarity Matrices

To address our research questions about the expected similarity of neural responses based on voice identity, we constructed several representational dissimilarity matrices (RDMs) that defined our study’s predictions. Each RDM had the same overall structure as the ONDM, with 276 unique cells defining predicted dissimilarity for a given pair of items in the experiment.

To test whether brain representations of voice identities align with a theoretical framework of telling together and telling apart, we constructed hypothetical model RDMs in which within-identity comparisons were coded as similar (0), and between-identity comparisons were coded as dissimilar (1). Seven such RDMs were constructed in order to inspect representations across all voices as well as specific voice pairings:

- Between-Speaker > Within-Speaker (All Voices): Familiar-Lab =1, Familiar-New = 1, Lab-New =1; Familiar-Familiar = 0, Lab-Lab = 0, New-New = 0)
- Between Speaker > Within-Speaker (Voice Pair): Familiar-Lab = 1, Familiar-Familiar = 0
- Between Speaker > Within-Speaker (Voice Pair): Familiar-Lab = 1, Lab-Lab = 0
- Between Speaker > Within-Speaker (Voice Pair): Familiar-New = 1, Familiar-Familiar = 0
- Between Speaker > Within-Speaker (Voice Pair): Familiar-New = 1, New-New = 0
- Between Speaker > Within-Speaker (Voice Pair): Lab-New = 1, Lab-Lab = 0
- Between Speaker > Within-Speaker (Voice Pair): Lab-New = 1, New-New = 0

To test whether brain representations of voice identities are shaped by speaker familiarity, we constructed hypothetical model RDMs in which within-identity comparisons were coded as more similar (lower values) depending on increasing familiarity. Four such RDMs were constructed, in order to inspect representations across all voices as well as specific voice pairings :

Within-speaker dissimilarity (All Voices) : Familiar-Familiar = 1; Lab-Lab = 2; New-New = 3

Within-speaker dissimilarity (Voice Pair) : Familiar-Familiar = 0; Lab-Lab = 1

Within-speaker dissimilarity (Voice Pair) : Familiar-Familiar = 0; New-New = 1

Within-speaker dissimilarity (Voice Pair) : Lab-Lab = 0; New-New = 1

For all hypothetical model RDMs, the diagonal and all non-relevant item comparisons were excluded from the analysis (i.e. coded with NaN). The final hypothetical model RDMs are available as open data(Open Science Framework: http://doi.org/10.17605/OSF.IO/QRZWG).

#### Acoustic model Representational Dissimilarity Matrices

As our focus was on the perceptual representation of voices rather than basic auditory processing, we constructed an additional acoustic representational dissimilarity matrix (RDM) per participant to account for the basic acoustic similarities of the voice stimuli. Acoustic dissimilarity was calculated by computing the Long-Term Average Spectra (LTAS) of voice samples using the *librosa* package within Python.^52^ For each voice stimulus (8 items x 3 identities per participant), we first performed a Short-Term Fourier Transform (STFT). Second, we averaged the power spectra obtained for all windows of the STFT across the time axis. Third, for all pairs of voice samples presented in a task to a participant, we calculated the cosine similarity between averaged power spectra. Finally, we deduced dissimilarity scores from the cosine similarities and compiled them in a matrix with dimensions matching the hypothetical model RDMs. The final acoustic model RDMs per participant are available as open data (Open Science Framework: http://doi.org/10.17605/OSF.IO/QRZWG).

#### RSA Searchlight mask

We conducted searchlight analyses within a pre-defined binarised mask, informed by previous work in the person identity perception literature. The mask was based on group-level probabilistic maps of face-selective, voice-selective, and multi-modal person-selective regions, based on functional localiser experiments run by Tsantani et al.,^20^ with a separate sample of participants. Voice-selective regions were identified by contrasting listeners’ neural responses to human (verbal and non-verbal) vocalisations compared to man-made or environmental sounds in two separate localiser tasks.^20,24^ These regions included bilateral superior temporal sulci (STS) and superior temporal gyri (STG), and the bilateral temporal voice areas (TVAs^24^), and encompassed primary auditory cortex in both hemispheres. Face-selective regions were identified by comparing neural responses to silent non-speaking videos of famous and non-famous faces to silent videos of moving natural or man-made objects. These regions comprised tissue within the right occipital gyrus (“occipital face area”/OFA) and the right fusiform gyrus (“fusiform face area”/FFA), as well as the right posterior STS. Multi-modal person-selective regions were established by comparing neural responses to audio-visual speaking clips of famous and non-famous people to audio-visual clips of moving human-made objects or natural scenes. These regions comprised the precuneus/posterior cingulate, frontal pole/superior frontal gyrus, and orbitofrontal cortex/ventromedial prefrontal cortex, and bilateral temporal poles/anterior inferior temporal cortex.

Using the imcalc tools in SPM, a probabilistic mask of each of the regions of interest from Tsantani et al.,^20^ was thresholded to include voxels present in the individual normalised masks of at least 10 participants (33.3%) in their sample. The final mask image was formed by summing the thresholded ROI images into a single image, binarising this combined image, and finally reslicing to voxel dimensions of 2 x 2 x 2mm to match the resolution of the current study’s EPI images. The final mask image is available as open data (Open Science Framework: http://doi.org/10.17605/OSF.IO/QRZWG).

For some participants with larger heads, the EPI data acquisition field of view failed to capture all voxels in the searchlight mask. This affected parts of the precuneus region of interest and, more rarely, the most posterior parts of the STG/STS regions, in a subset of participants. The CosMoMVPA toolbox accounts for missing voxels by adjusting the degrees of freedom in statistical tests, such that group results could be reported for the full searchlight mask. A heatmap of coverage across the 26 participants is included as open data (Open Science Framework: http://doi.org/10.17605/OSF.IO/QRZWG).

#### Exploratory univariate analysis

An exploratory univariate analysis was conducted in SPM12 in order to compare the magnitude of the BOLD response to the three voice identities in the experiment. For this, the preprocessed fMRI data were smoothed using a Gaussian kernel of 6mm full width at half maximum (FWHM). For each participant, a univariate general linear model was then constructed and estimated in SPM12. All experimental and null trial onsets, as well as trial responses, were modelled as instantaneous events and convolved with the canonical haemodynamic response function. The 8 experimental items per voice were modelled as a single regressor per voice and per run. The null events, responses, and movement parameters were modelled as for the RSA analysis (see imaging pre-processing). To account for participants missing some voxels in the searchlight mask, implicit masking was removed by setting the threshold to -Inf for model estimation. Contrast estimate maps were calculated per participant for each voice condition versus null baseline (averaged across all runs), as well as for Familiar > Lab [1 -1], Familiar > New [1 -1], and Lab > New [1 -1].

Four group models were estimated in SPM12:

- One-way within subjects ANOVA: This included the per-participant contrast images Familiar > Baseline, Lab > Baseline, and New > Baseline. A Main Effect of Voice Condition was estimated using the contrast [1 -1 0; 0 1 -1].
- One-sample t-test Familiar vs Lab: This included the per-participant contrast image Familiar > Lab. The effect Familiar > Lab was estimated using the contrast [1], and Lab > Familiar estimated using the contrast [-1].
- One-sample t-test Familiar vs New: This included the per-participant contrast image Familiar > New. The effect Familiar > New was estimated using the contrast [1], and New > Familiar estimated using the contrast [-1].
- One-sample t-test Lab vs New: This included the per-participant contrast image Lab > New. The effect Lab > New was estimated using the contrast [1], and New > Lab estimated using the contrast [-1].

All group models included an explicit mask comprising the same searchlight mask as applied for RSA. All group results are displayed and reported at a voxel height threshold of p <.05 with familywise error correction for multiple comparisons. Brain data were visualised and anatomically labelled using Mango (Research Imaging Institute, UTHSCSA). Please see Figure S1 and Table S3.

## Supplementary Material

Supplemental information

## Data Availability

Data used to generate the figures have been deposited via the Open Science Framework (http://www.doi.org/10.17605/OSF.IO/QRZWG). The data are publicly available as of the date of publication. To protect participant anonymity, raw MRI and speech audio data are not shared. Code used to generate the figures has been deposited via the Open Science Framework (http://www.doi.org/10.17605/OSF.IO/QRZWG). The files are publicly available as of the date of publication. Any additional information required to reanalyze the data reported in this paper is available from the lead contact upon request.

## Highlights

- Recognition of personally familiar voices is robust to within-speaker variation
- Brain response patterns to these voices show higher within-speaker dissimilarity
- This suggests finer-grained representations of personally familiar speech
- The findings complement reports of familiar-talker intelligibility benefits

## In brief

Kanber et al. report that brain responses to personally familiar voices are more dissimilar across stimuli than responses to lab-trained or unfamiliar voice identities. The findings suggest that extensive and varied exposure to personally familiar voices results in the development of finer-grained neural representations of those voices.

## References

[1] Scott S, McGettigan C, Matsumoto D, Hwang HC, Frank MG (2016). APA Handbook of Nonverbal Communication APA Handbooks in psychology®.

[2] Lavan N, Burton AM, Scott SK, McGettigan C (2019). Flexible voices: Identity perception from variable vocal signals. Psychon Bull Rev.

[3] Johnson J, McGettigan C, Lavan N (2020). Comparing unfamiliar voice and face identity perception using identity sorting tasks. Q J Exp Psychol (Hove).

[4] Kanber E, Lavan N, McGettigan C (2022). Highly accurate and robust identity perception from personally familiar voices. J Exp Psychol Gen.

[5] Lally C, Lavan N, Garrido L, Tsantani M, McGettigan C (2023). Neural representations of naturalistic person identities while watching a feature film. Imaging Neurosci.

[6] Lavan N, Burston LF, Ladwa P, Merriman SE, Knight S, McGettigan C (2019). Breaking voice identity perception: Expressive voices are more confusable for listeners. Q J Exp Psychol (Hove).

[7] Lavan N, Merriman SE, Ladwa P, Burston LFK, Knight S, McGettigan C (2020). ‘Please sort these voice recordings into 2 identities’: Effects of task instructions on performance in voice sorting studies. Br J Psychol.

[8] Lavan N, Knight S, Hazan V, McGettigan C (2019). The effects of high variability training on voice identity learning. Cognition.

[9] Njie S, Lavan N, McGettigan C (2023). Talker and accent familiarity yield advantages for voice identity perception: A voice sorting study. Mem Cognit.

[10] Smith HMJ, Baguley TS, Robson J, Dunn AK, Stacey PC (2019). Forensic voice discrimination by lay listeners: The effect of speech type and background noise on performance. Appl Cogn Psychol.

[11] Stevenage SV, Tomlin R, Neil GJ, Symons AE (2021). May I Speak Freely? The Difficulty in Vocal Identity Processing Across Free and Scripted Speech. J Nonverbal Behav.

[12] Mike Burton A (2013). Why has research in face recognition progressed so slowly? The importance of variability. Q J Exp Psychol (Hove).

[13] Lavan N, Burston LFK, Garrido L (2019). How many voices did you hear? Natural variability disrupts identity perception from unfamiliar voices. Br J Psychol.

[14] Kriegeskorte N, Mur M, Bandettini PA (2008). Representational similarity analysis - connecting the branches of systems neuroscience. Front Syst Neurosci.

[15] Domingo Y, Holmes E, Johnsrude IS (2020). The benefit to speech intelligibility of hearing a familiar voice. J Exp Psychol Appl.

[16] Holmes E, Domingo Y, Johnsrude IS (2018). Familiar Voices Are More Intelligible, Even if They Are Not Recognized as Familiar. Psychol Sci.

[17] Holmes E, Johnsrude IS (2020). Speech spoken by familiar people is more resistant to interference by linguistically similar speech. J Exp Psychol Learn Mem Cogn.

[18] Johnsrude IS, Mackey A, Hakyemez H, Alexander E, Trang HP, Carlyon RP (2013). Swinging at a Cocktail Party: Voice Familiarity Aids Speech Perception in the Presence of a Competing Voice. Psychol Sci.

[19] Formisano E, De Martino F, Bonte M, Goebel R (2008). “Who” Is Saying “What”? Brain-Based Decoding of Human Voice and Speech. Science.

[20] Tsantani M, Kriegeskorte N, McGettigan C, Garrido L (2019). Faces and voices in the brain: A modality-general person-identity representation in superior temporal sulcus. NeuroImage.

[21] Awwad Shiekh Hasan B, Valdes-Sosa M, Gross J, Belin P (2016). “Hearing faces and seeing voices”: Amodal coding of person identity in the human brain. Sci Rep.

[22] Belin P, Fecteau S, Bédard C (2004). Thinking the voice: neural correlates of voice perception. Trends Cogn Sci.

[23] Maguinness C, Roswandowitz C, von Kriegstein K (2018). Understanding the mechanisms of familiar voice-identity recognition in the human brain. Neuropsychologia.

[24] Belin P, Zatorre RJ, Lafaille P, Ahad P, Pike B (2000). Voice-selective areas in human auditory cortex. Nature.

[25] Belin P, Zatorre RJ, Ahad P (2002). Human temporal-lobe response to vocal sounds. Brain Res Cogn Brain Res.

[26] Kriegstein KV, Giraud A-L (2004). Distinct functional substrates along the right superior temporal sulcus for the processing of voices. NeuroImage.

[27] Roswandowitz C, Kappes C, Obrig H, von Kriegstein K (2018). Obligatory and facultative brain regions for voice-identity recognition. Brain.

[28] Schall S, Kiebel SJ, Maess B, von Kriegstein K (2015). Voice Identity Recognition: Functional Division of the Right STS and Its Behavioral Relevance. J Cogn Neurosci.

[29] von Kriegstein K, Eger E, Kleinschmidt A, Giraud AL (2003). Modulation of neural responses to speech by directing attention to voices or verbal content. Brain Res Cogn Brain Res.

[30] Zäske R, Awwad Shiekh Hasan B, Belin P (2017). It doesn’t matter what you say: FMRI correlates of voice learning and recognition independent of speech content. Cortex.

[31] Andics A, McQueen JM, Petersson KM, Gál V, Rudas G, Vidnyánszky Z (2010). Neural mechanisms for voice recognition. NeuroImage.

[32] Belin P, Zatorre RJ (2003). Adaptation to speaker’s voice in right anterior temporal lobe. NeuroReport.

[33] Bethmann A, Scheich H, Brechmann A (2012). The Temporal Lobes Differentiate between the Voices of Famous and Unknown People: An Event-Related fMRI Study on Speaker Recognition. PLoS One.

[34] Birkett PB, Hunter MD, Parks RW, Farrow TF, Lowe H, Wilkinson ID, Woodruff PW (2007). Voice familiarity engages auditory cortex. NeuroReport.

[35] Campanella S, Belin P (2007). Integrating face and voice in person perception. Trends Cogn Sci.

[36] Shah NJ, Marshall JC, Zafiris O, Schwab A, Zilles K, Markowitsch HJ, Fink GR (2001). The neural correlates of person familiarity. A functional magnetic resonance imaging study with clinical implications. Brain.

[37] Luthra S (2021). The Role of the Right Hemisphere in Processing Phonetic Variability Between Talkers. Neurobiol Lang (Camb).

[38] Kriegeskorte N, Goebel R, Bandettini P (2006). Information-based functional brain mapping. Proc Natl Acad Sci USA.

[39] Davis MH, Johnsrude IS (2003). Hierarchical processing in spoken language comprehension. J Neurosci.

[40] Hickok G, Poeppel D (2007). The cortical organization of speech processing. Nat Rev Neurosci.

[41] Rauschecker JP, Scott SK (2009). Maps and streams in the auditory cortex: nonhuman primates illuminate human speech processing. Nat Neurosci.

[42] Scott SK, Blank CC, Rosen S, Wise RJ (2000). Identification of a pathway for intelligible speech in the left temporal lobe. Brain.

[43] Scott SK, Johnsrude IS (2003). The neuroanatomical and functional organization of speech perception. Trends Neurosci.

[44] Holmes E, Johnsrude IS (2021). Speech-evoked brain activity is more robust to competing speech when it is spoken by someone familiar. NeuroImage.

[45] Kreiman J, Sidtis D (2011). Foundations of Voice Studies.

[46] Lavan N, McGettigan C (2023). A model for person perception from familiar and unfamiliar voices. Commun Psychol.

[47] Sidtis D, Kreiman J (2012). In the beginning was the familiar voice: Personally familiar voices in the evolutionary and contemporary biology of communication. Integr Psychol Behav Sci.

[48] Tsantani M, Kriegeskorte N, Storrs K, Williams AL, McGettigan C, Garrido L (2021). FFA and OFA encode distinct types of face identity information. J Neurosci.

[49] Holmes E, To G, Johnsrude IS (2021). How Long Does It Take for a Voice to Become Familiar? Speech Intelligibility and Voice Recognition Are Differentially Sensitive to Voice Training. Psychol Sci.

[50] Yan X, Volfart A, Rossion B (2023). A neural marker of the human face identity familiarity effect. Sci Rep.

[51] Lavan N, Knight S, McGettigan C (2019). Listeners form average-based representations of individual voice identities. Nat Commun.

[52] McFee B, Raffel C, Liang D, Ellis DPW, McVicar M, Battenberg E, Nieto O (2015). librosa: Audio and music signal analysis in Python.

[53] Hazan V, Baker R (2011). Acoustic-phonetic characteristics of speech produced with communicative intent to counter adverse listening conditions. J Acoust Soc Am.

[54] R Core Team (2023). R: A Language and Environment for Statistical Computing.

[55] Anwyl-Irvine AL, Flitton A, Kirkham N, Evershed JK (2020). Gorilla in our midst: An online behavioral experiment builder. Behav Res Methods.

[56] Brainard DH (1997). The Psychophysics Toolbox. Spat Vis.

[57] Bates D, Mächler M, Bolker B, Walker S (2015). Fitting Linear Mixed-Effects Models Using lme4. J Stat Soft.

[58] Lenth RV (2024). emmeans: Estimated Marginal Means, aka Least-Squares Means. Version R package version 1100.

[59] Moeller S, Yacoub E, Olman CA, Auerbach E, Strupp J, Harel N, Ugvurbil K (2010). Multiband multislice GE-EPI at 7 tesla, with 16-fold acceleration using partial parallel imaging with application to high spatial and temporal whole-brain fMRI. Magnetic resonance in medicine.

[60] Xu J, Moeller S, Auerbach EJ, Strupp J, Smith SM, Feinberg DA, Ugvurbil K (2013). Evaluation of slice accelerations using multiband echo planar imaging at 3 T. Neuroimage.

[61] Oosterhof NN, Connolly AC, Haxby JV (2016). CoSMoMVPA: Multi-Modal Multivariate Pattern Analysis of Neuroimaging Data in Matlab/GNU Octave. Front Neuroinform.

[62] Garrido L, Vaziri-Pashkam M, Nakayama K, Wilmer J (2013). The consequences of subtracting the mean pattern in fMRI multivariate correlation analyses. Front Neurosci.

[63] Ramírez FM (2017). Representational confusion: the plausible consequence of demeaning your data. bioRxiv.

[64] Smith SM, Nichols TE (2009). Threshold-free cluster enhancement: addressing problems of smoothing, threshold dependence and localisation in cluster inference. NeuroImage.

