# Supplementary material for: Representations of personally familiar voices are better resolved in the brain
Source: Curr Biol. Author manuscript; Available in PMC 2025 Sep 16. (PMC7618125; doi:10.1016/j.cub.2025.03.081)
Supplement: Supplemental information [file EMS208281-supplement-Supplemental_information.zip › 1-s2.0-S0960982225004282-mmc1.pdf]

Current Biology, Volume *35*

Supplemental Information

Representations of personally familiar voices are better resolved in the brain

Elise Kanber, Clare Lally, Raha Razin, Victor Rosi, Lúcia Garrido, Nadine Lavan, and Carolyn McGettigan

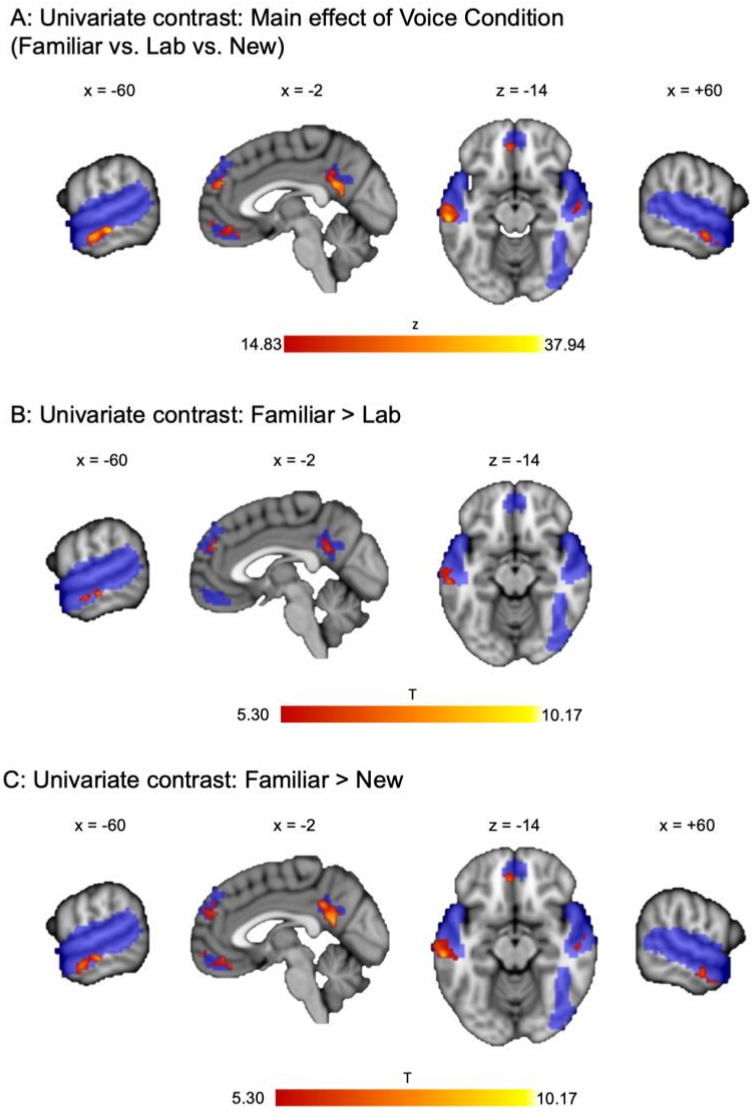

Figure S1. Results of an exploratory univariate analysis comparing the magnitude of the average response to the three voice identities, Related to Table S3 and STAR Methods. Significant activations exceed a voxel height threshold of $p < .05$ (FWE-corrected). Blue shading indicates the searchlight mask of face-, voice-, and person-selective brain regions of interest. Coordinates are shown in Montreal Neurological Institute (MNI) stereotactic space.

Comparison		Peak Anatomical Label	Cluster size (mm^3)	Peak x	Peak y	Peak z	Statistic (TFCE-corrected z)
Between-Speaker > Within-Speaker (All Voices)	(Familiar-Lab & Familiar-New & Lab-New) > (Familiar-Familiar & Lab-Lab & New-New)	Left superior temporal gyrus	33096	-64	-48	22	3.72
		Right superior temporal gyrus	21656	56	-56	10	3.43
		Medial frontal gyrus	6696	2	44	38	2.97
		Cingulate gyrus	2280	0	-58	32	2.60
		Cingulate gyrus	592	-10	-52	32	2.25
		Anterior cingulate	512	0	44	-14	2.29
		Right inferior frontal gyrus	496	50	16	-10	2.15
		Left superior temporal gyrus	184	-48	18	-30	2.41
Between-Speaker > Within-Speaker (Voice Pairs)	Familiar-Lab > Lab-Lab	Left inferior parietal lobe	44992	-48	-40	30	3.72
		Right superior temporal gyrus	33776	48	-46	20	3.72
		Medial frontal gyrus	8808	-6	44	38	3.43
		Cingulate gyrus	4520	0	-54	30	3.19
		Right fusiform gyrus	1952	44	-48	-10	2.30
		Right inferior temporal gyrus	1920	52	-72	0	2.24
		Anterior cingulate	1848	-2	44	-12	2.34
	Familiar-New > New-New	Left inferior parietal lobe	51888	-48	-40	30	3.72
		Right inferior parietal lobe	40072	54	-46	24	3.72
		Superior frontal gyrus	8856	-4	48	48	3.35
		Right fusiform gyrus	6808	42	-52	-12	2.60
		Posterior cingulate	4800	2	-54	26	2.93
		Anterior cingulate	1808	-2	44	-12	2.39
Between-Speaker < Within-Speaker (Voice Pairs)	Familiar-Lab < Familiar-Familiar	Left superior temporal gyrus	2512	-52	6	-12	-2.64
		Left superior temporal gyrus	1088	-42	-46	4	-2.40
		Left superior temporal gyrus	1056	-60	-26	0	-2.66
		Left superior temporal gyrus	208	50	-54	6	-2.07
	Lab-New < Lab-Lab	Right middle occipital gyrus	624	36	-86	-6	-2.33
		Right culmen	528	40	-52	-20	-2.46

Table S1. Comparing between-speaker ("telling apart") and within-speaker ("telling together") dissimilarity in the brain responses to voices, Related to Figure 2.

Table includes all significant clusters exceeding 20 voxels ($160mm^3$).

Comparison		Peak Anatomical Label	Cluster size (mm^3)	Peak			Statistic (TFCE-corrected z)
				x	y	z	
Within-Speaker (All Voices)	Familiar-Familiar vs. Lab-Lab vs. New-New	Left inferior parietal lobe	43048	-48	-40	30	-3.72
		Right insula	27208	50	-40	20	-3.54
		Superior frontal gyrus	8752	-10	48	48	-3.43
		Right fusiform gyrus	5704	42	-52	-12	-3.04
		Cingulate gyrus	1696	-2	-56	30	-2.26
		Precuneus	864	-8	-50	36	-2.31
Within-Speaker (Voice Pairs)	Familiar-Familiar > Lab-Lab	Left superior temporal gyrus	42152	-50	-52	22	-3.72
		Right superior temporal gyrus	26680	56	-28	-2	-3.54
		Superior frontal gyrus	8320	-4	46	40	-3.43
		Right fusiform gyrus	2624	44	-48	-10	-2.73
		Posterior cingulate	1000	-2	-56	26	-2.69
	Familiar-Familiar > New-New	Left superior temporal gyrus	43520	-48	-40	30	-3.72
		Right inferior parietal lobe	29272	52	-24	-2	-3.72
		Superior frontal gyrus	8776	-10	48	48	-3.43
		Right fusiform gyrus	6128	42	-54	-12	-3.04
		Cingulate gyrus	2072	-2	-56	30	-2.31
		Precuneus	928	-16	-46	36	-2.41

Table S2. Comparing within-speaker ("telling together") dissimilarity in the brain responses to voices of differing familiarity, Related to Figure 3.

Table includes significant clusters exceeding 20 voxels ($160mm^3$).

Model	Contrast	Peak Anatomical Label	Cluster size (mm^3)	Peak x	Peak y	Peak z	Statistic (F/T)
Within-subjects one-way ANOVA	Main Effect of Voice Condition	Medial frontal gyrus	2344	6	50	16	37.94
		Middle temporal gyrus	2264	-62	-24	-14	34.79
		Posterior cingulate	1136	-2	-50	24	32.06
		Left middle temporal gyrus	712	60	-8	-18	23.94
		Anterior cingulate	488	-6	40	-12	25.76
		Left superior temporal gyrus	480	-50	-56	26	27.59
		Superior frontal gyrus	344	-10	44	48	21.59
		Medial frontal gyrus	248	0	60	-6	29.21
One-sample T-test	Familiar > Lab	Left middle temporal gyrus	784	-60	-24	-12	7.14
		Medial frontal gyrus	472	-10	52	20	7.19
		Cingulate gyrus	456	0	-46	32	6.35
		Medial frontal gyrus	392	8	50	16	7.94
		Left superior temporal gyrus	176	-48	-56	26	5.95
	Familiar > New	Medial frontal gyrus	2928	6	50	16	10.17
		Posterior cingulate	1904	-4	-54	18	8.90
		Left middle temporal gyrus	1904	-62	-24	-14	8.95
		Right middle temporal gyrus	688	56	-14	-20	7.42
		Anterior cingulate	512	-6	40	-12	7.71
		Left middle temporal gyrus	296	-38	-58	24	6.83

Table S3. Results of an exploratory univariate analysis comparing the magnitude of the average response to the three voice identities, Related to Figure S1 and STAR Methods.

Table includes significant clusters exceeding 20 voxels (160mm^3).
